# Navigating autonomy: unraveling the dual influence of job autonomy on workplace well-being in the gig economy

**DOI:** 10.3389/fpsyg.2024.1369495

**Published:** 2024-07-25

**Authors:** Zhitao Wan, Lei Zhang, Li Wang, Fang Zhang

**Affiliations:** ^1^School of Management, Guizhou University of Commerce, Guiyang, China; ^2^School of Business Administration, Huaqiao University, Quanzhou, China; ^3^College of Inland Open Economics, Guizhou University of Commerce, Guiyang, China

**Keywords:** job autonomy, positive emotion, work alienation, perceived algorithmic control, workplace well-being, gig economy

## Abstract

**Introduction:**

With the rapid rise of the gig economy globally, its characteristics of promoting employment and facilitating autonomy have supported its rapid growth and development in China. While the flexibility of gig work offers more employment options and income sources for workers, it also caused many problems and uncertainties. Workplace well-being is an important psychological factor that indicates the psychological state of workers and significantly predicts their behavior at work. However, previous studies on the gig economy rarely analyze gig workers’ workplace well-being, which is of great significance to improving their individual emotions, promoting their physical and mental health, and maintaining the sustainable development of the gig economy and society in general.

**Methods:**

This study draws on the cognitive-affective processing system framework to construct a moderated dual-mediator model to explore the dual influence mechanism of job autonomy on gig workers’ workplace well-being. Based on the data of 442 digital gig workers who were mainly engaged in manual labor.

**Results:**

The survey results show that job autonomy positively affects employees’ workplace well-being, and work alienation and positive emotion mediate this relationship. Perceived algorithmic control can moderate not only the influence of job autonomy on work alienation and positive emotion but also the indirect impact of job autonomy on workplace well-being through work alienation and positive emotion.

**Discussion:**

The finding of this research contributes to expand the comprehension of the relationship between gig-worker job autonomy and workplace wellbeing and this relationship’s underlying mechanism, holding significant implications for management practice.

## 1 Introduction

In recent years, the gig economy has flourished, and a large number of gig workers have achieved employment through online labor platforms. According to a public report by Ministry of Human Resources and Social Security of the People’s Republic of China, the number of flexible employment employees has reached 200 million, and the number of gig workers in China will reach 400 million in 2036, accounting for half of China’s employment population (Guo et al., 2023). Gig platforms such as Meituan Takeaway, Ele.me, and Didi have attracted a large number of gig workers and become dynamic and mobile symbols of value in cities (Pei et al., 2021a). In the algorithm management environment, there are differences in the perception of job characteristics of gig workers. Some individuals are concerned about job security, while others are concerned about job autonomy (Felix et al., 2023). Since gig workers engage in gig work through self-employment, and there is no affiliation relationship between practitioners and platforms (Guo et al., 2023). Meanwhile, online labor platform are mostly based on the working mode of online order acceptance and offline service, gig workers can freely decide the online and offline time and work autonomy (Duggan et al., 2020). Compared with traditional employment methods, the platform does not impose mandatory requirements on job attendance and on-duty time, and the self-employed work mode is more flexible and free (Zhang and Yang, 2022), and empowered workers to independently allocate time and energy (Wood et al., 2019),which has become one of the important factors attracting gig workers (Deng and Li, 2021). Therefore, gig work is emerging topic in the field of work autonomy. However, the regulation of the labor process of gig workers by algorithmic technology will encourage gig workers to increase their working time input without autonomy, resulting in the “autonomy-control” paradox (Putnam et al., 2014; Shevchuk et al., 2019). In gig context, the labor experience and work attitude of platform workers will show unique characteristics (Huang, 2019). With the expansion of the scale of gig workers, the experience of practitioners is receiving increasing attention.

Job characteristics can have behavioral and psychological effects. As a core incentive, job autonomy refers to the flexibility of working hours and the autonomy of workers over the content and scheduling of their work (Wheatley, 2017), which has significant psychological impacts on workers. As a job characteristic, numerous studies have confirmed the impact of job autonomy on positive emotions and intrinsic motivation (Hackman and Oldham, 1976), job burnout (Park et al., 2014), decent work perception and voice behavior (Kao et al., 2022). However, the gig economy differs from the traditional organization model and provides a new perspective for researching organizational behavior. Under the gig economy, the traditional employment model between enterprises and employees is broken, and gig workers are faced with a working environment that differs significantly from that of traditional workers. The dynamic interaction between gig workers and the platform is “gig platform–gig worker–customer” (Long et al., 2021). By receiving orders and working on the online labor platform, workers can freely decide the working time, place, rest and vacation, and even determine the labor supply and salary level (Wu and Li, 2018). It also enables gig workers to freely choose transitional employment between full-time work, temporary work or retirement (Donovan et al., 2016), which effectively improves the job autonomy of gig workers. Nevertheless, existing research on the impact of job autonomy on workers’ mental states and behavior has focused on the traditional employment mode, with gig workers remaining understudied. Job autonomy is a typical characteristic of gig workers (Sundararajan, 2016). In the gig context, since the huge difference between the Online Labor Platform and the traditional employment organization in the control of the labor process, gig workers can freely decide the working time, place, rest time, and even determine the labor supply and salary level on the platform, which gives the work autonomous (Wu and Li, 2018). Meanwhile, online labor platforms use algorithmic technology to implicitly regulate the labor process and compress the time for gig workers to complete tasks. Meanwhile, by increasing the reward for their work, gig workers are induced to work more hours to earn more money (Ran and Zhao, 2023), and achieve “soft control” over gig workers (Shapiro, 2018), leading to the “autonomy-control” paradox of workers with work autonomy showing longer working hours instead (Putnam et al., 2014). Therefore, despite the autonomy of gig workers’ work, under the gig context, gig workers’ labor experience and working attitude will show unique characteristics (Huang, 2019). Workplace well-being describes the well-being of employees in the workplace (Warr, 1987), which refers to the positive evaluation and positive emotion of individuals on their work, which mainly comes from the satisfaction brought by the ability improvement, value realization of individuals in the process of completing task (Zheng et al., 2015). As an important psychological factor, Workplace well-being is significantly correlated with employees’ work behaviors and results (Wright and Cropanzano, 2004), and its importance in workplace is increasingly prominent (Singhal and Rastogi, 2018). Workplace well-being among gig workers will directly influences the healthy development of gig platforms. Existing studies on job autonomy and workplace well-being are all based on traditional employment models (Daniels and Guppy, 1994; Li et al., 2015), however, few studies have yet explored the perception of job autonomy of gig workers in the gig economy and its impact. Therefore, based on the gig economy, it is necessary to explore the impact of job autonomy of gig workers on their behavior and cognition in the algorithmic control context. In addition, previous studies have explored Work engagement (Bakker et al., 2014), work motivation (Demerouti and Bakker, 2011), job crafting (Slemp et al., 2015), intrinsic motivation (Nie et al., 2015) play a mediating role in the relationship between job autonomy and employee well-being. However, these studies are all based on a single cognitive perspective to explore the relationship between the two. Therefore, this study adopts the cognitive-affective processing system framework developed by Mischel and Shoda (1995) to investigate the logical relationship between job autonomy and workplace well-being and systematically reveal the mechanism of job autonomy’s influence on workplace well-being. This framework holds that individual behavior is affected by individual emotional and cognitive processing of external information. Emotional and cognitive processing often interact to affect the individual’s response to external information and behavior. Specifically, according to this framework, the external environmental characteristics of an individual can activate the cognitive and affective in the personality system, thus significantly impacting individual behavior. Meanwhile, individual traits can interact with situational characteristics and drive differences in the cognitive, affective, and behavioral performance of individuals.

First, job autonomy affects gig workers’ workplace well-being by influencing cognitive changes. The sense of meaninglessness and powerlessness due to alienation from work diminishes volition and self-control by exhausting individual psychological capital (Inzlicht and Schmeichel, 2012), which can be predicted by job characteristics. Meanwhile, previous research results also show that work alienation can negatively affect employees’ job satisfaction (Lagios et al., 2022), and job satisfaction is significantly positively correlated with workplace well-being (Zheng et al., 2015). Therefore, this study proposes the cognitive path of “job autonomy→work alienation→workplace well-being.”

**Figure 1:**
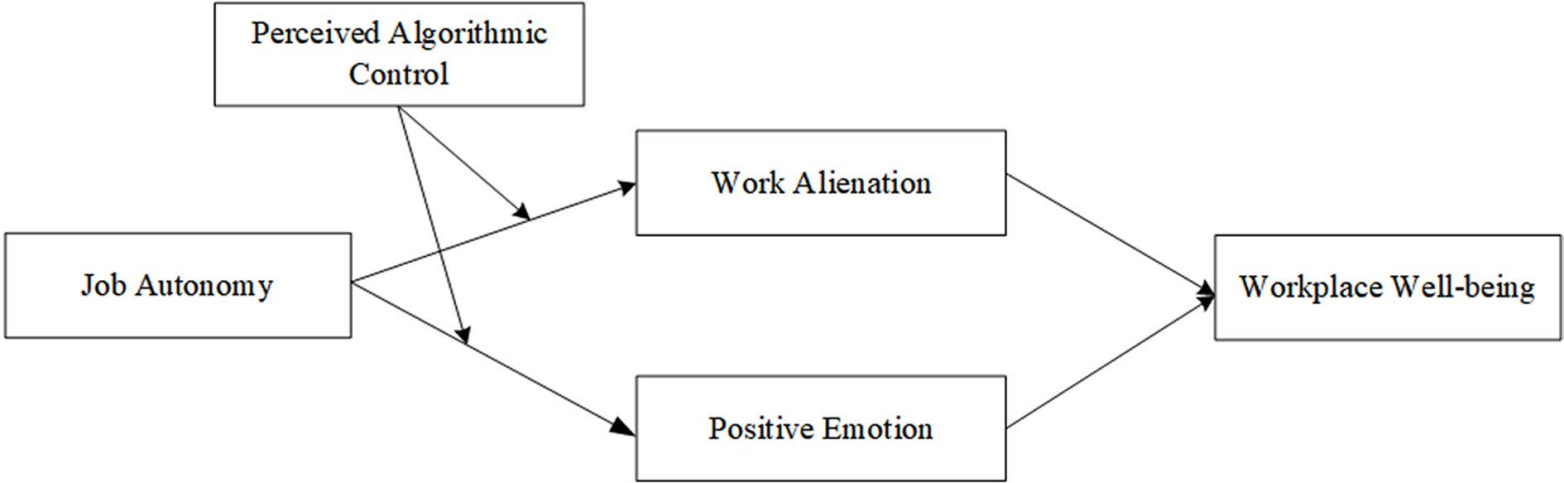
Theoretical model.

Second, job autonomy may also affect workplace well-being by triggering emotional changes in gig workers. Job characteristics can significantly predict employees’ emotions (Xu et al., 2023), thus influencing their well-being through their emotion. Therefore, this study proposes the emotion path of “job autonomy→positive emotion→workplace well-being.” In addition, the above cognitive-emotional response processes may also vary with each individual, i.e., be influenced by individual characteristics. Since the interaction between gig workers and platforms differs significantly from that between employees and individual organizations under the traditional employment model, algorithmic management has replaced traditional organization management to become the main way of managing the labor process of gig workers (Chen, 2020). Gig workers’ different perceptions of algorithmic control affect their attitudes and behaviors (Lee, 2018). When workers believe that the purpose of algorithmic control is to monitor and deter misconduct, they become stressed. When facing pressure, individuals may make differentiated evaluations according to specific situations and their cognitive feelings, which mainly include challenging and obstructing (Pei et al., 2021a). Although stressors make individuals feel pressured, they tend to believe that their interests will be enhanced and consolidated after overcoming them, and then they will adopt positive strategies to deal with them. In contrast, obstructive stressors will hinder the realization of individual work goals and career development. Therefore, in the light of the cognitive-affective processing system framework, this research explores the indirect effect of job alienation and positive emotions in the mutual influence between job autonomy and job well-being and examines the regulating role of gig workers’ algorithm-based perception in this relationship (theoretical model see Figure 1).

## 2 Literature review and hypotheses

### 2.1 Job autonomy and workplace well-being

In the context of the gig economy, the development of Internet information technology has provided workers with more flexibility and freedom of autonomy in terms of working time and location. This prominent employment feature of gig work is a significant attractor for a large number of gig workers. The decision-making system and flexible work design of the gig platform provide autonomous support for gig workers (Behl et al., 2021), and gig workers can independently decide working hours, working places, working methods, etc. They also can choose to work on multiple platforms simultaneously to reduce their dependence on any one platform (Sundararajan, 2016). Meanwhile, gig workers can freely arrange their working hours and choose the type of work they want to do based on the business operation architecture and customer information resources designed by the application without being controlled and constrained by the employer (Thomas, 2018).This flexibility and autonomous working atmosphere satisfy the autonomy needs of individuals and further enhance their degree of self-determination and autonomy cognition (Chen et al., 2022). This sense of control over the work environment satisfies workers’ need for autonomy and perceived self-competence and also enhances their awareness of these factors.

As a work resource, job autonomy is the degree of control over job decision-making authority (Ohly et al., 2006). It helps workers develop a stronger sense of control in the pursuit of career goals. Employees who are granted more autonomy act according to their own wishes at work, which means taking responsibility for work-related outcomes while also improving their productivity and intrinsic motivation (Langfred and Moye, 2004). The current study holds that job autonomy positively impacts workplace well-being.

First, job autonomy improves employees’ control over work tasks and provides them with more resources to complete work under freer working conditions (Xu et al., 2023). Autonomy leads to more positive emotions and intrinsic motivation (Hackman and Oldham, 1976), resulting in higher job satisfaction.

Second, work resources will lead to “resource gain,” which will promote the learning, growth and development of workers. Job autonomy offers workers discretion and the ability to independently decide how to carry out their work, allowing them to take responsibility for the results of their work. Therefore, job autonomy can enrich work experience, improve problem-solving ability and innovation (Hu and Mao, 2019), and enhance workers’ sense of autonomy and competence, thus improving their workplace well-being. Finally, studies on the gig economy have found that workers who participate in platform work still report higher levels of well-being after accounting for differences in income and that flexible work and full discretion over working hours are the main drivers of gig workers’ job satisfaction (Berger et al., 2019). Thus, the above analytical logic leads us to propose the following hypothesis:

*H*1: Job autonomy will be positively related to the workplace well-being of gig workers.

### 2.2 The negative effect of job autonomy on workplace well-being: the cognitive path of work alienation

Work alienation refers to the psychological experience that an individual perceives a separation between himself and his work (Banai et al., 2004). Individual perceptions of job characteristics can significantly predict work alienation. Compared with the strict discipline and continuous high-intensity production mode of traditional factory work, gig platform has no mandatory requirements for on-duty and on-duty time, and can independently decide working hours, working places and working methods, with high work autonomy (Zhang and Yang, 2022). It is an important factor to attract gig workers (Chen, 2020). In the light of the conservation of resource theory, individuals tend to strive to acquire and maintain resources that they deem valuable. As a work resource, job autonomy can motivate individuals and help them cope with work effectively (Ohly et al., 2006). Autonomy can bring more resources to individuals, improve employees’ work motivation, increase work input, and trigger the process of incentive gain (Li et al., 2015), help employees successfully achieve work goals, motivate employees to learn and grow, and promote employees’ development (Bakker et al., 2007). Improving employees’ sense of self-determination and competence is conducive to alleviating employees’ sense of work alienation (Huang and Chen, 2017). If the algorithmic management of the platform is at the expense of the interests and freedom of gig workers, it will reduce their autonomy, lead to the loss of resources, and if it is not supplemented in time, it will enter the state of work alienation. At the same time, work alienation, as a negative factor in the organization, has a negative impact on employees’ work behavior and attitude.

Meanwhile, as a negative factor in the organization, work alienation has a negative impact on employees’ work-related behaviors and attitudes. Existing research shows that work alienation will be significant negatively related to employees’ job involvement, organizational commitment, career commitment and overall job satisfaction (Hirschfeld et al., 2000). Employees who feel alienated from their work develop a sense of social isolation and become cognitively distanced from work, reducing their identification with work and its meaningfulness, thus weakening their internal motivation to complete work tasks. Alienated workers view remuneration as the only purpose of their work (Ohly et al., 2006), which can make them feel strongly dominated and controlled by work, feel helpless and empty, become slaves and vassals of work, and think that even life has no value (Huang and Chen, 2017a). In addition, work alienation triggers the interpersonal needs of employees, prompting them to strengthen their connection with the outside world using the Internet as a source of emotional stability and belonging, thus reducing their attention to work and negatively affecting their workplace well-being. Thus, the above analytical logic leads us to propose the following hypothesis:

*H*2: Work alienation has an indirect effect in the mutual influence between gig workers’ job autonomy and workplace well-being.

### 2.3 The positive effect of job autonomy on workplace well-being: emotion path of positive emotion

Emotions are responses to environmental events perceived as beneficial or harmful, which involves a complex process of conceptualization and evaluation (Halbesleben et al., 2014). Job characteristics are important antecedents that influence an individual’s psychological state at work and, thus, their work outcomes. Positive emotion is a psychological state in which individuals experience positive emotions such as happiness, pride and excitement (Watson et al., 1988). Job autonomy evokes positive emotions, leading to more positive experiences.

First, job autonomy can effectively enhance one’s sense of control at work, which is conducive to reducing stress. When an employee’s sense of control at work increases, autonomy leads to more positive emotions and intrinsic motivation (Hackman and Oldham, 1976).

Second, the flexibility and autonomy of the time, space, and content arrangement of gig jobs can effectively alleviate workplace stress and anxiety and reduce the occupational mental health problems of workers (Farfán et al., 2020). In addition, job autonomy allows employees to decide when and how to respond to work requirements, indicating that employees have better resources and freer working conditions (Xu et al., 2023), which can cushion the pressure and exhaustion brought by work requirements and reduce individual physical and mental consumption. Meanwhile, job autonomy can ensure that employees have enough resources to process work information and absorb and transform it into their own resources, which is beneficial for employees to recover psychological resources (Wu and Guo, 2022). Workers can use these resources to prevent the transformation of stress into anxiety and other negative emotions (Zhang and Yan, 2020), thus enhancing work resources and promoting the generation of positive emotions.

Emotions significantly predict an individual’s perceptual and behavioral abilities and attention span (Fredrickson, 2001). Individuals immersed in negative emotions are more inclined to engage in negative cognitive evaluation, which reduces workplace well-being. In contrast, emotions can expand the scope of individual attention and cognition, and the more positive emotions an individual has, the more energy, focus and pleasure they experience (Watson et al., 1988). By expanding individual cognition and motivation, positive emotions can improve people’s enthusiasm and activity ability, increase their optimism and confidence, and enable them to devote themselves to work more passionately (Wang and Wang, 2021). Under such circumstances, individuals can participate in their work more positively and feel that the time and energy they invest in their work will be meaningfully rewarded (Hu and Mao, 2019), enhancing their workplace well-being.

Meanwhile, emotional responses affect individuals’ cognitive judgment of the working environment by acting on their memory and understanding, which affects their job satisfaction (Williams et al., 1996). Individuals with negative emotions tend to focus on unpleasant people and things and adopt a negative cognitive processing mode, so they have low job satisfaction. However, positive emotions help individuals to extract positive information from memory and interpret work events and environments more positively, which is conducive to the formation of job satisfaction, which improves workplace well-being (Choi et al., 2011). In addition, positive emotions help individuals obtain lasting resources, such as social support, and promote good interpersonal interactions (Zhang and Yan, 2020). Such interpersonal interactions can be used to cope with the depletion of resources under work pressure and buffer its effect, which helps employees maintain a positive psychological state, thus enhancing workplace well-being. Thus, the above analytical logic leads us to propose the following hypothesis:

*H*3: Positive emotion has an indirect effect in the mutual influence between gig workers’ job autonomy and workplace well-being.

### 2.4 Moderating role of perceived algorithmic control

An algorithm is usually defined as a sequence of steps that converts input data into a desired output. Thus, algorithmic management decisions can be automatically calculated according to an analysis and decision model, and then the platform workers carry out and complete the work according to the resulting operation result (Schildt, 2017). The interaction between gig workers and gig platforms is markedly different from that between individuals and organizations in the traditional employment model: gig platforms primarily manage gig workers’ labor processes algorithmically using technology (Chen, 2020), providing them with a work environment that provides autonomy and control (Waldkirch et al., 2021). How do gig workers perceive the interaction between them and platforms and recognize and evaluate algorithmic control, which will fundamentally affect or shape their attitudes and behaviors. Individuals have differences in their understandings of algorithmic control, causing their reactions to it will differ accordingly (Pei et al., 2021a).

First, regarding cognition, gig workers who perceive low algorithmic control in their work tend to take the instructions generated by algorithm management, including embedded rating and reputation information, as decision information and optimize the work process and work results accordingly (Chen et al., 2022), which can enhance their sense of autonomy. Meanwhile, gig workers who perceive low algorithmic control believe that their behavior is the result of independent choice and control, which promotes the formation of internal motivation. As an individual’s desire to devote energy to an assignment stem from their interest and enjoyment of that assignment (Gagné and Deci, 2005), increased motivation will enhance employees’ perception of meaning in and connection with their work. Thus, gig workers with low algorithmic control perception can feel more autonomy, further enhancing the negative relationship between job autonomy and work alienation. On the contrary, gig workers who perceive high algorithmic control will perceive the gig platform as manipulative (Chen et al., 2022), and be more sensitive to the platform’s “panoramic prison” management (Kellogg et al., 2020), feel more closely monitored and constrained, leading their work autonomy is greatly dissolved (Wood et al., 2019). Meanwhile, gig workers with high algorithmic control perceive will show resistance to the algorithm (Kellogg et al., 2020), making gig workers self-depletion for a long time, consuming a large amount of psychological resources, increase the difficulty of workers to complete the work, resulting the decline of workers’ motivation and the increase of job burnout, inducing work alienation (Li et al., 2015). In addition, gig workers with high algorithmic control perceive will have complex, opaque and dehumanized interpretations of algorithm management, resulting in a sense of procedural injustice and negative experience of the work environment (Lee, 2018; Pei et al., 2021b), thus damaging individuals’ perception of their own competence (Kim and Beehr, 2020), resulting in lower autonomy, thus weakening the inhibitory effect of job autonomy on work alienation.

Second, regarding emotions, the main reason why gig workers choose to work on gig platforms is the flexibility and autonomy these platforms offer (Sundararajan, 2016). Gig workers who perceive strong algorithmic control will see a gap between their ideal and reality, creating self-cognition conflict, which induces negative emotions. Meanwhile, ambiguous platform algorithm management reduces gig workers’ perceived transparency of task assignments. Thus, gig workers who perceive high algorithmic control will perceive their task assignment as unjust (Pei et al., 2021b), and procedural injustice is an important cause of relative deprivation, which leads to negative emotions (Wei et al., 2022). Meanwhile, because workers with high algorithmic perception feel the pressure information from the algorithm management on the task timeliness, panoramic supervision and penalty mechanism, negative emotions will also be stimulated (Liu et al., 2023). Since an increase in negative emotions leads to decreased positive emotions (Cacioppo and Berntson, 1994), thus undermining the positive relationship between job autonomy and positive emotions. On the contrary, when gig workers perceive low algorithmic control, the characteristics of their job align with their psychological expectations, the consistency of self-cognition is preserved, and negative emotions are decreased, thus effectively maintaining their emotional stability. Thus, the above analytical logic leads us to propose the following hypothesis:

*H*4: Perceived algorithmic control negatively moderates the negative impact of job autonomy on work alienation.

*H*5: Perceived algorithmic control negatively moderates the positive impact of job autonomy on positive emotion.

The above hypotheses suggest that work alienation and positive emotion play an indirect role in the interaction between the dual paths of job autonomy affecting workplace well-being. In view of this, this study further proposes the moderated mediation effect hypothesis, that is, the dual path through which gig workers’ job autonomy affects their workplace well-being through work alienation and positive emotions will be moderated by their perceived algorithmic control. Higher perceived algorithmic control among gig workers predicts a weaker mediating role of work alienation and positive emotions. Thus, the above analytical logic leads us to propose the following hypothesis:

*H*6: The interactive effect of job autonomy and perceived algorithmic control on workplace well-being is mediated by work alienation, such that mediated effect of job autonomy on workplace well-being through work alienation is weaker when perceived algorithmic control is high rather than low.

*H*7: The interactive effect of job autonomy and perceived algorithmic control on workplace well-being is mediated by positive emotions, such that mediated effect of job autonomy on workplace well-being through positive emotions is weaker when perceived algorithmic control is high rather than low.

## 3 Methods

### 3.1 Sample and procedure

This study focuses on service-oriented digital gig workers who perform physical labor tasks assigned to them on location-based digital platforms. This includes couriers and ride-service workers using e-hailing but excludes knowledge workers who receive work via web-based digital platforms. E-hailing drivers and takeaway riders are the most active groups in the gig economy (Pei et al., 2021a). They interact closely with algorithms. The work process is always subject to algorithm standardization, processes, quotas, and high-intensity real-time control (Lee et al., 2015). In addition to their strong algorithmic control over their labor process (Lee et al., 2015), another major reason lies in their work autonomy (Duggan et al., 2020). In the process of work, E-hailing drivers and takeaway riders are allowed to invest their own means of production in work, carry out their labor based on the information and instructions from the platform, and their income mainly depends on the number of orders they receive. They are free to decide when to go online and how long to stay online (Wu and Li, 2018), which makes their work autonomous. Therefore, this study selected e-hailing drivers and couriers in Guiyang as study subjects.

In this study, 162 junior students majoring in business administration at a western university were invited to serve as contacts to collect questionnaires in exchange for credits. Students participated in offline surveys by recruiting E-hailing drivers and takeaway riders. The students handed out 600 questionnaires to the E-hailing drivers and takeaway riders. To improve the recovery rate of questionnaire, the researchers have repeatedly emphasized that the survey data were for academic research purposes only and is apply for overall relationship analysis, and assured them that they would remain anonymous. Meanwhile, research participation will be incentivized by offering students course credit, after their recruited respondents carefully fill out the questionnaire.

We distributed a total of 600 questionnaires, and 442 valid after excluding those that answered incomplete questions, or provided repeated answers. The effective recovery rate is 73.67%. Males accounted for 219 participants (49.5%). Most participants were 26–35 years old, with 172 (38.9%). Most participants had completed junior college or obtained a bachelor’s degree, accounting for 69.9%. full-time gig workers accounted for 67.4%, and part-time gig workers accounted for 32.6%. In terms of years spent as a gig worker, 26.2% were gig workers for less than 1 year, 41.6% for 2 to 4 years, 19.5% for 5 to 7 years, and 3.6% for more than 11 years. In terms of average monthly earnings, most of gig workers earn a monthly income of 4,001–6,000 yuan, accounting for 39.4%. Most gig workers raise one child, accounting for 36.9%. Most gig workers are married, accounting for 52.5%. Sample information are presented in Table 1.

**Table 1:** Sample information (*N* = 442).

Variable	Classify	Frequency	Percent (%)	Variable	Classify	Frequency	Percent (%)
Gender	Female	223	50.5	Age	≤25	162	36.7
	Male	219	49.5		26 ~ 35	172	38.9
Education	High school degree or below	108	24.4		36 ~ 45	86	19.5
	Junior college degree	129	29.2		≥46	22	5.0
	Bachelor degree	180	40.7	Tenure	≤1	116	26.2
	Master degree or above	25	5.7		2 ~ 4	184	41.6
Participate in gig work	Full-time	298	67.4		5 ~ 7	86	19.5
	Part-time	144	32.6		8 ~ 10	40	9.0
Marital status	Married	210	47.5		≥11	16	3.6
	Spinsterhood	232	52.5	Average monthly	≤2000	42	9.5
Number of children	Not raising children	150	33.9		2001 ~ 4000	133	30.1
	Raising a child	163	36.9		4,001 ~ 6,000	174	39.4
	Raising two child	80	18.1		6001 ~ 8000	56	12.7
	Raising 3 or more children	49	11.1		>8000	37	8.4

### 3.2 Measures

In this research, mature measure scales were adopted to assess all variables, and these original scale in English were translated into Chinese on the basis of a back-translation procedure (Brislin, 1970). This research adopts five-point scales to measure the above dimensions (1 = “strongly disagree” and 5 = “strongly agree”), except for control variables.

#### 3.2.1 Job autonomy

A 3-item scale developed by Spreitzer (1995) was adopted. Sample items included “The work schedule is largely up to the worker.” Cronbach’s Alpha was 0.88.

#### 3.2.2 Work alienation

We assessed work alienation with measures recommended by Nair and Vohra (2009), which is divided into eight items in two dimensions: personal alienation and social alienation. Sample items include “I do not feel connected to the events in my workplace.” Cronbach’s Alpha was 0.88.

#### 3.2.3 Positive emotion

Positive emotions were assessed using the Panas Mood Scale recommended by Watson et al. (1988) and combined with the studies of Zhang and Yan (2020). Five items were selected to measure happiness, enthusiasm, activeness, pride, and inspiration. Cronbach’s Alpha was 0.85.

#### 3.2.4 Perceived algorithmic control

We assessed algorithmic control perception with measures recommended by Pei et al. (2021a) for gig workers, which consists of 11 items in three dimensions: normative guidance, tracking evaluation, and behavior constraint. Sample items include, “The platform uses algorithms to intelligently assign my work tasks,” “The platform uses algorithms to continuously track my work progress,” and “The platform uses algorithms to provide me with cash rewards to motivate me to work hard.” Cronbach’s Alpha was 0.87.

#### 3.2.5 Workplace well-being

A 6-item scale recommended by Zheng et al. (2015) based on the organizational context in China was adopted. Sample items include “Work is meaningful experience for me.” Cronbach’s Alpha was 0.86.

#### 3.2.6 Control variables

With reference to the existing literature, gender, age, education, participate in gig work (full-time/part-time), tenure of gig workers, average monthly earnings, marital status and number of children were controlled to fully reveal the role of the core variables.

### 3.3 Statistical analysis

SPSS 23.0 was adopted to test descriptive statistics and correlation analysis of the main variables, and Mplus 7.4 was adopted to test confirmatory factor analysis on five major variables. The macro program PROCESS of SPSS 23.0 was used for hypothesis testing, and bootstrap sampling times were 5,000.

## 4 Results

### 4.1 Confirmatory factor analysis

We adopt a CFA to examine our measurement model, including job autonomy, positive emotion, work alienation, perceived algorithmic control and workplace well-being, to judge whether discriminative validity among the core variables meet the standard. As indicated in Table 2. the five-factor model has a better fit than other models, *χ^2^ = 839.96, df = 485, CFI = 0.94, TLI = 0.94, RMSEA = 0.04, SRMR = 0.04*. This indicate that the five core constructs of this study had a good discrimination validity.

**Table 2:** Results of CFA.

Model	χ^2^	df	CFI	TLI	SRMR	RMSEA
Five-factor: JA; PAC; WA; PE; WWB	839.96	485	0.94	0.94	0.04	0.04
Four-factor: JA; PAC; WA + PE; WWB	1755.30	489	0.79	0.77	0.10	0.08
Three-factor: JA + PAC; WA + PE; WWB	2376.47	492	0.68	0.66	0.11	0.09
Two-factor: JA + PAC; WA + PE + WWB	3333.56	494	0.52	0.49	0.15	0.11
One-factor: JA + WA + PE + PAC + WWB	4310.12	495	0.35	0.31	0.15	0.13

*N* = 442. JA, job autonomy; PAC, perceived algorithmic control; WA, work alienation; PE, positive emotion; WWB, workplace well-being.

### 4.2 Descriptive analysis

Means, standard deviations, and correlation of the variable in this research are presented in Table 3. Job autonomy positively impacted workplace well-being (*r* = 0.34, *p* < 0.01) and negatively impacted work alienation (*r* = −0.16, *p* < 0.01); Job autonomy positively related to positive emotion (*r* = 0.20, *p* < 0.01); Work alienation negatively related to workplace well-being (*r* = −0.21, *p* < 0.01); Positive emotion positively related to workplace well-being (*r* = 0.30, *p* < 0.01).

**Table 3:** Summary statistics and intercorrelations.

Variable	M	SD	1	2	3	4	5	6	7	8	9	10	11	12
1. Gender	1.50	0.50												
2. Age	1.93	0.87	−0.20^**^											
3. Education	2.28	0.90	0.07	−0.16^**^										
4. Tenure	2.22	1.05	−0.21^**^	0.66^**^	−0.14^**^									
5. Participate in gig work	1.33	0.47	0.17^**^	−0.29^**^	0.06	−0.28^**^								
6. Average monthly earnings	2.80	1.05	−0.21^**^	0.30^**^	0.05	0.43^**^	−0.33^**^							
7. Number of children	1.06	0.98	−0.10^*^	0.58^**^	−0.21^**^	0.53^**^	−0.16^**^	0.17^**^						
8. Marital status	1.52	0.50	0.16^**^	−0.66^**^	0.23^**^	−0.52^**^	0.29^**^	−0.24^**^	−0.66^**^					
9. Job autonomy	3.22	0.75	0.07	0.01	0.14^**^	0.02	0.06	0.07	0.00	−0.00				
10. Perceived algorithmic control	3.28	0.61	0.01	0.01	0.09	0.03	0.07	0.01	0.01	−0.03	0.36^**^			
11. Work alienation	3.12	0.74	−0.08	0.01	−0.00	0.04	0.09^*^	0.00	0.04	0.00	−0.16^**^	0.07		
12. Positive emotion	3.13	0.76	0.01	0.02	0.11^*^	0.02	0.02	−0.04	0.03	−0.02	0.20^**^	0.19^**^	0.01	
13. Workplace well-being	3.25	0.72	−0.02	0.04	0.12^*^	0.03	−0.02	0.03	−0.01	−0.03	0.34^**^	0.34^**^	−0.21^**^	0.30^**^

*N* = 442, ^*^p < 0.05, ^**^p < 0.01.

### 4.3 Hypothesis testing

**Figure 2:**
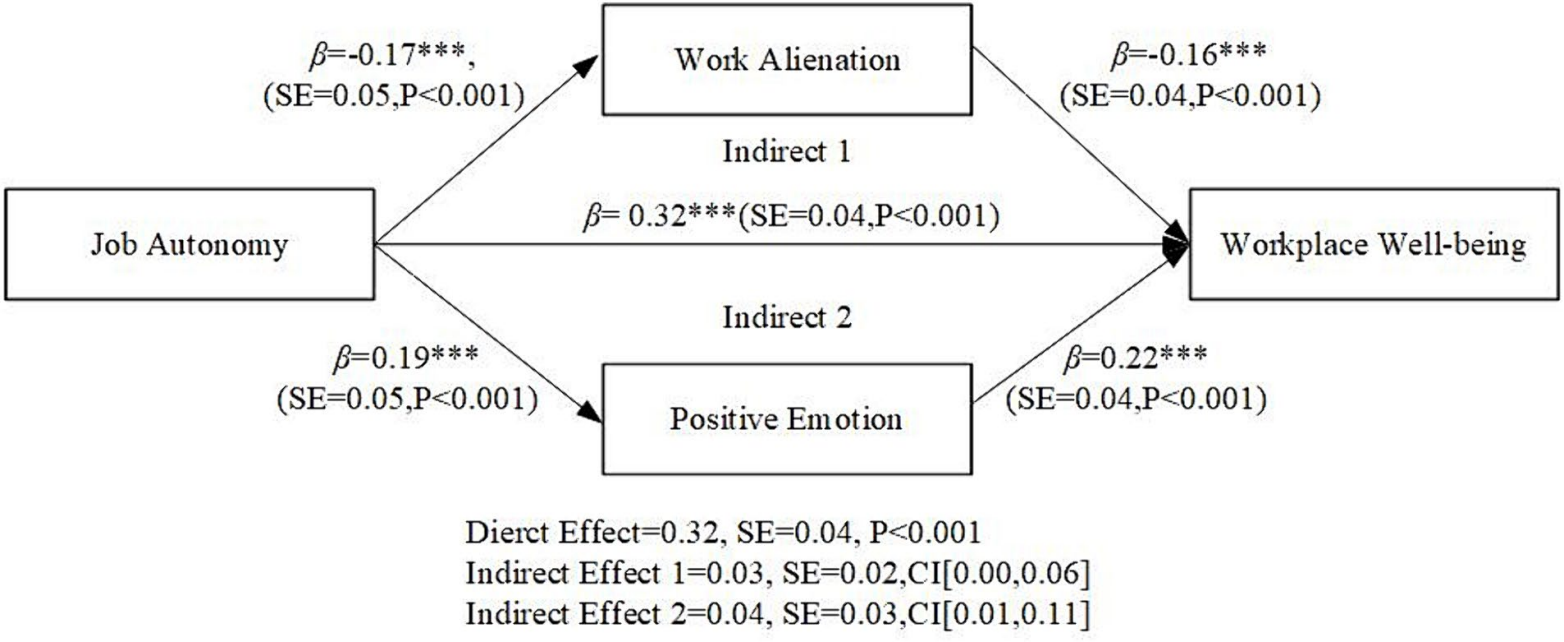
Structural equation model test results.

In this study, SPSS 23.0 statistical software was used for hypothesis testing. Specifically, job autonomy was taken as the independent variable; work alienation and positive emotion as mediating variables; workplace well-being as the dependent variable; and gender, age, education, full-time/part-time, tenure and marital status as the control variable. Bootstrapping was run 5,000 times to examine the mediating effect. The test results of relevant structural equation models are shown in Figure 2. For details see Table 4. Job autonomy positively impacted workplace well-being (*β* = 0.32, *p* < 0.001, *M2*), supporting H1. Job autonomy was significantly associated with work alienation (*β* = −0.17, *p* < 0.001, *M5*) and positive emotion (*β* = 0.19, *p* < 0.001, *M8*), respectively. When job autonomy, work alienation and positive emotion jointly predicted workplace well-being, although the impact of job autonomy on workplace well-being was lower than M2, it was remained significant (*β = 0.24*, *p < 0.001, M3*). Meanwhile, work alienation was negatively related to workplace well-being (*β = −0.17*, *p < 0.001, M3*) and the positive influence of positive emotions on workplace well-being was still significant (*β = 0.23*, *p < 0.001, M3*). These results support H2 and H3. In addition, we conduct the bootstrap method suggested by Hayes (2013) to further verify the indirect effect. The results show that the mediating effects of work alienation and positive emotion between job autonomy and workplace well-being were 0.03 and 0.04, respectively. The 95% bias-corrected bootstrap confidence interval were [0.00, 0.06] and [0.01, 0.11], respectively—excluding zero. These results indicated that work alienation and positive emotion play a mediating role in these relationships. Thus, H2 and H3 was further supported.

**Table 4:** Regression results for hypothesis testing.

Variable	Workplace well-being			Work alienation			Positive emotion		
	M1	M2	M3	M4	M5	M6	M7	M8	M9
Gender	−0.02	−0.06	−0.07	−0.14	−0.13	−0.13	0.01	−0.02	−0.00
Age	0.03	0.03	0.03	−0.02	−0.02	−0.02	0.01	0.01	0.01
Education	0.11^**^	0.07	0.06	0.01	0.03	0.02	0.11**	0.09*	0.08*
Tenure	0.01	0.00	0.00	0.04	0.04	0.05	0.03	0.03	0.01
Full-time / part-time	0.00	−0.04	−0.01	0.19	0.21	0.17	0.02	−0.01	0.01
Average monthly earnings	−0.00	−0.02	−0.00	0.00	0.01	0.03	−0.05	−0.06	−0.07
Number of children	−0.04	−0.04	−0.04	0.04	0.04	0.05	0.02	0.02	0.02
Marital status	−0.10	−0.08	−0.07	0.04	0.03	0.07	−0.05	−0.04	−0.05
Job autonomy		0.32^***^	0.24^***^		−0.17^***^	−0.19^***^		0.19^***^	0.13*
Work alienation			−0.17^***^						
Positive emotion			0.23^***^						
Perceived algorithmic control						0.16^**^			0.16^**^
Job autonomy * Perceived algorithmic control						0.20^***^			−0.21^***^
R^2^	0.02	0.13	0.21	0.02	0.05	0.10	0.02	0.06	0.10
∆R^2^		0.10	0.08		0.03	0.05		0.04	0.05
F	1.19	6.89	10.12	1.27	2.52	4.34	1.14	2.85	4.52

*N* = 442, ^*^p < 0.05, ^**^p < 0.01, ^***^p < 0.001. The non-standardized regression coefficients are reported in the table.

**Figure 3:**
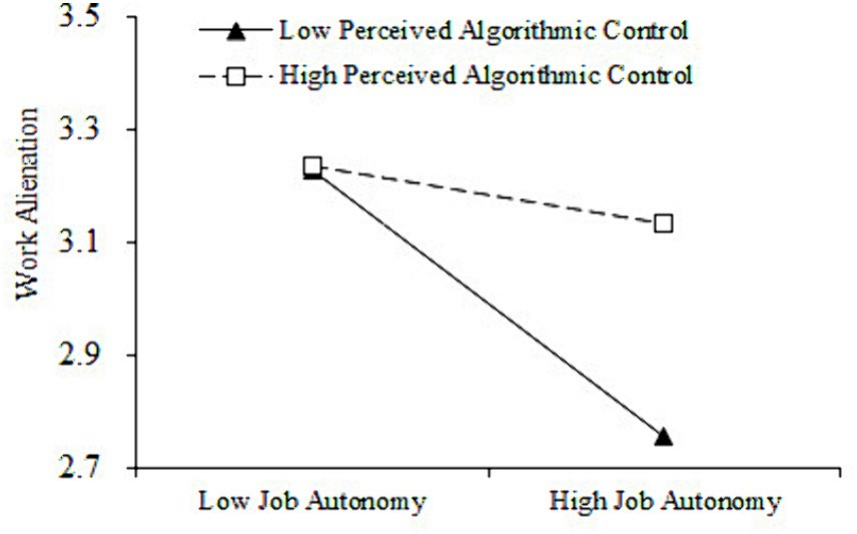
Moderating effect of perceived algorithmic control between job autonomy and gig workers’ work alienation.

**Figure 4:**
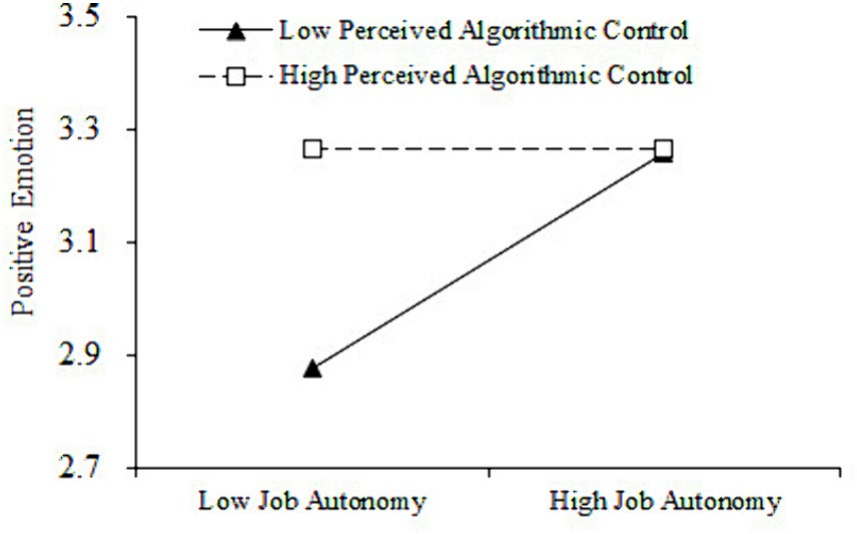
Moderating effect of perceived algorithmic control between job autonomy and gig workers’ positive emotion.

The interaction terms of job autonomy and perceived algorithmic control were reconstructed through centralized processing to verify the moderating role of perceived algorithmic control. As indicated in Table 4, the interaction term of job autonomy and perceived algorithmic control was positively related to work alienation (*β = 0.20*, *p < 0.001*, *M6*), which indicates that perceived algorithmic control play a significant regulating role in the interaction between job autonomy and work alienation. This study further conducted a simple slope test and plotted the adjustment effect. As shown in Figure 3, when perceived algorithmic control was low, job autonomy was negatively related to work alienation but relatively strong (*β = −0.31*, *p < 0.001*). However, when perceived algorithmic control was high, job autonomy was still negatively correlated with work alienation and relatively weak, but not significant (*β = −0.07*, *p > 0.05*). Therefore, H4 was further supported. In addition, As indicated in Table 3, the interaction term of job autonomy and perceived algorithmic control was negatively related to positive emotion (*β = −0.21*, *p < 0.001*, *M9*), prove that perceived algorithmic control play a regulating role in the interaction between job autonomy and positive emotion. As indicated in Figure 4, the simple slope test results show that when perceived algorithmic control was low, job autonomy was positively related to positive emotion but relatively strong (*β = 0.25*, *p < 0.05*). On the contrary, when perceived algorithmic control was high, job autonomy relatively weak related to positive emotion, but not significant (*β = 0.00*, *p > 0.05*). Therefore, H5 was further supported.

According to the suggestion of Edwards and Lambert (2007), we applied macro program PROCESS to further examine whether perceived algorithmic control can moderate the mediating role of work alienation and positive emotion. As indicated in Table 5. When perceived algorithmic control was low, the mediating role of work alienation played in interaction between job autonomy and workplace well-being was significant (*β* = 0.06, 95% CI = [0.02, 0.11], excluding 0). In addition, when perceived algorithmic control as high, the mediating role of work alienation played in interaction between job autonomy and workplace well-being not significant (*β* = 0.01, 95% *CI* = [−0.02, 0.06], including 0). Meanwhile, when perceived algorithmic control varied from the above one standard deviation below the mean to below one standard deviation below the mean, the indirect effects were also significant (difference value −0.05, 95% *CI* = [−0.11, −0.01], excluding 0). Therefore, H6 was supported. When perceived algorithmic control was low, the mediating role of positive emotion played in interaction between job autonomy and workplace well-being was significant (*β* = 0.05, 95% *CI* = [0.01, 0.12], excluding 0). In addition, the indirect effect not significant when perceived algorithmic control was high (*β* = 0.00, 95% *CI* = [−0.05, 0.05], including 0). Meanwhile, when perceived algorithmic control varied from the above one standard deviation below the mean to below one standard deviation below the mean, the indirect effects were also significant (difference value was −0.05, 95% *CI* = [−0.12, −0.00], excluding 0). Thus, H7 was supported.

**Table 5:** Bootstrap result of moderated mediation effect.

Path	Effect	SD	95% CI
1. Job autonomy→work alienation→workplace well-being			
Low perceived algorithmic control (-1SD)	0.06	0.02	[0.02, 0.11]
High perceived algorithmic control (+ 1SD)	0.01	0.02	[−0.02, 0.06]
Dif(*Δ*)	−0.05	0.02	[−0.11, −0.01]
2. Job autonomy→positive emotion→workplace well-being			
Low perceived algorithmic control (-1SD)	0.05	0.03	[0.01, 0.12]
High perceived algorithmic control (+ 1SD)	0.00	0.02	[−0.05, 0.05]
Dif(*Δ*)	−0.05	0.03	[−0.12, −0.00]

Bootstrap = 5000.

## 5 Conclusion and discussion

### 5.1 Conclusion

This study draws on the cognitive-affective processing system framework to examine the impact of job autonomy on the workplace well-being of gig workers and examine the mediating effect of work alienation and positive emotion, as well as the regulating effect of perceived algorithmic control. Through the analysis of 442 questionnaire responses from e-hailing drivers and couriers, we found that job autonomy was positively related to workplace well-being. The mediation effect of work alienation and positive emotion between job autonomy and workplace well-being was significant. Perceived algorithmic control positively regulated the impact of job autonomy on work alienation and positive emotion. Meanwhile, the mediating relationship between job autonomy and workplace well-being through work alienation and positive emotion was regulated by perceived algorithmic control.

### 5.2 Theoretical contributions

First, this study extends the research scope of workplace well-being. The existing researches mainly focus on the factors that affect the workplace well-being under the traditional employment model (Chughtai et al., 2015; Zhang et al., 2020; Wu et al., 2023). The online labor platform strengthens the control of the labor process through algorithm technology, and the difference between workers’ autonomy restricted in behavior and enhanced autonomy in subjective perception has gradually attracted the attention of the academic community. It is very important to deeply analyze the impact of algorithmic management of online labor platform on work autonomy (Liu et al., 2021). This study examined the effect of gig job autonomy on workplace well-being. Existing studies have shown that under the management of algorithms, gig workers will automatically extend their working hours, resulting in increased labor intensity (Petriglieri et al., 2019), turnover (Ramsay et al., 2000), and triggering work–family conflict (Shevchuk et al., 2019), while paying less attention to the impact on workplace well-being. This study focuses on the possible impact of job autonomy on gig workers’ workplace well-being under algorithmic control, which is helpful for a more comprehensive understanding of the impact of job autonomy paradox on individual psychology under the context of gig economy.

Second, this study analyzed and tested the process mechanism of job autonomy’s influence on gig workers’ workplace well-being and revealed the cognitive and emotional response process of gig workers to this mechanism, which is conducive to a more comprehensive understanding of the influence of gig job autonomy on workplace well-being. Previous research on organizational behavior has focused on the mediating mechanism among variables, and the exploration of the intermediary mechanism can reveal the process of motivation, cognition, and psychological change generated by individual behavior (Zhou et al., 2021). Current studies have explored the mechanism of influence of gig worker’s job autonomy on work results from a single perspective, such as emotional exhaustion (Ogbonnaya et al., 2017) and identity strain (Liu et al., 2021). It is not possible to fully explain the influence mechanism of job autonomy on gig workers’ workplace well-being. Therefore, this study drew on a cognitive-affective processing system framework to construct a moderated dual-mediator model to explore the dual influence mechanism of job autonomy on employees’ workplace well-being. This study’s findings indicate that although job autonomy, as a work resource, can improve the workplace well-being of gig workers, the mechanism underlying this interaction may be complicated. Under the cognitive path, the workplace well-being of gig workers is due to the interpretation and meaning construction of the characteristics of job autonomy. Taking job autonomy as an important resource can effectively help them cope with work effectively to motivate themselves, enhance their sense of work meaning, reduce their sense of work alienation, and weaken their sense of cognitive distance from work. Under the emotional path, job autonomy enhances the sense of control at work, reduces workplace stress, alleviates occupational mental health problems, suppresses negative emotions caused by job requirements, encourages positive cognitive processing, and thus improves well-being at work. Therefore, by examining the mediating role of work alienation and positive emotions, this paper helps to reveal the cognitive and emotional changes of job autonomy in the context of the gig economy on the workplace well-being of gig workers and deepens the understanding of the impact of gig workers’ job autonomy on workplace well-being.

Finally, this study provides theoretical support for the effect of algorithmic management on the cognitive changes and emotional responses of gig workers. Most of the existing researches discuss the influence of algorithm management through qualitative method, and there is a lack of empirical analysis (Liu et al., 2022). This study explores the moderating effect of gig workers’ algorithm-controlled perception, builds a cognitive-affective dual path theoretical model, and further defines the boundary conditions of the mediating mechanism in the relationship between job autonomy and workplace well-being through their cognitive and affective paths. Thus, this research deepens the cognition of the mechanism and boundary conditions of gig job characteristics on gig workers’ workplace well-being.

### 5.3 Practical implications

Gig workers operate in a working environment that differs from that of regular employees in traditional organizations, the algorithmic management adopted by online labor platforms could threaten the health of gig platforms. Therefore, combined with the management practice of platform algorithmic control, the exploration of the influence of gig workers’ job autonomy on the workplace well-being has certain practical and guiding significance for improving the workplace well-being of gig workers.

First, job autonomy increases gig workers’ workplace well-being. However, the algorithm management of the Online Labor Platform will reduce the control of gig workers’ work and have an inhibiting effect on gig worker’s well-being. Therefore, through algorithm design, online labor platforms can develop gamed task allocation methods and humanized operating systems to enhance the perception of work pleasure and sense of achievement in labor process, and alleviate the pressure of algorithm control on gig workers. Secondly, differences in perception of algorithmic control will affect gig workers’ cognition of job autonomy, and algorithmic transparency can alleviate the negative effects of algorithmic control (Pei et al., 2024). Therefore, the platform should strengthen the training of gig workers, disclose the function and decision-making process of the algorithm system to gig workers, improve their understanding of the algorithm system, promote positive perceptions of algorithm control among gig workers, enhance the perception of job autonomy, and stimulate workplace well-being.

Second, this study found that work alienation and positive emotions have a conductive effect on job autonomy and workplace well-being. Therefore, online labor platforms can more effectively promote the positive impact of job autonomy on gig workplace well-being through emotional and cognitive guidance. For positive emotions, online labor platforms can strengthen humanistic care for gig workers, formulate reasonable and moderate algorithmic assessment standards, and avoid emotional exhaustion caused by high performance assessment that causes gig workers to fall into high-intensity labor. In addition, the platform can enrich the application of game functions in the work situation, improve the fun of performing tasks, create a more relaxed and pleasant working atmosphere, and stimulate the positive emotions of gig workers. For job alienation, platforms should provide gig workers with career development services, enhance gig workers’ sense of control over their future work. Meanwhile, strengthen the technical support and real-time information feedback of algorithm management, enhance the work ability and confidence of gig workers, and enhance the work value of gig workers. Beyond that platform can strengthen the monitoring of gig workers’ psychological, pay attention to daily psychological counseling and management. Thereby reducing job alienation and promoting gig workers’ workplace well-being.

### 5.4 Limitations and future research directions

Although this paper has generated meaningful research results, it had some limitations.

First, there are different types of gig jobs. However, this study included only e-hailing drivers and couriers, professions with low registration thresholds and low professional requirements, failing to show the overall phenomenon of gig workers in the gig economy. Future studies can include online crowdsourcing workers and consultants in professional fields as research subjects, expand the sample group, and explore the behavioral and cognitive differences of different types of gig workers in the same working environment.

Second, the research gathered cross-sectional data, which cannot explain the causal relationship between the study variables. Longitudinal tracking or experimental research can be used to help reveal the causal relationship between the study variables. Meanwhile, the participants in this study were all from the Guiyang area, which could decrease the findings’ generalizability. Therefore, the collection of data should be expanded to improve the external validity of research conclusions.

Third, according to the cognitive-affective theoretical framework, this paper explores the indirect effect of work alienation and positive emotions in work autonomy and workplace well-being among gig workers. The mediating role of cognitive and affective factors such as self-efficacy and gratitude can be further explored to reveal the intrinsic relationship between them. Although this study generated discussion on the moderating effect of perceived algorithmic control, differences in workers’ cognition and behavior are affected not only by individual differences but also by situational factors, such as culture and policy. Therefore, future studies should strengthen the influence of situational factors to more systematically reveal the boundary conditions of this relationship.

## Data availability statement

The raw data supporting the conclusions of this article will be made available by the authors, without undue reservation.

## Ethics statement

The survey process and procedures used in this study adhere to the tenets of the Declaration of Helsinki. Ethics approval was obtained from the Academic Committee at Guizhou University of Commerce. The participants provided their written informed consent to participate in this study.

## Author contributions

ZW: Formal analysis, Funding acquisition, Project administration, Writing – original draft, Writing – review & editing. LZ: Conceptualization, Writing – review & editing. LW: Data curation, Investigation, Writing – review & editing. FZ: Data curation, Writing – original draft.
