# Supplementary material for: Navigating autonomy: unraveling the dual influence of job autonomy on workplace well-being in the gig economy
Source: Front Psychol. 2024 Jul 25;15:1369495. doi: 10.3389/fpsyg.2024.1369495 (PMC11307207; doi:10.3389/fpsyg.2024.1369495)
Supplement: Supplementary file 1 [file Table_1.DOCX]

Supplementary Material

# Appendix A: Measurement Concept and Code

Appendix A. Measurement Concept and Code

| Variables | Item# | Measure | Code |
| --- | --- | --- | --- |
| Job autonomy  (JA) | JA1 | In most ways my life is close to my ideal. | Using with a five-point Likert scale, Variable’s value is calculated on the mean value from these items. |
|  | JA2 | The conditions of my life are excellent. |  |
|  | JA3 | I am satisfied with my life. |  |
| Positive emotion  (PE) | PE1 | During my work, I often feel happy. | Using with a five -point Likert scale, Variable’s value is calculated on the mean value from these items. |
|  | PE2 | During my work, I often feel full of enthusiasm. |  |
|  | PE3 | During my work, I am always active. |  |
|  | PE4 | During my working life, I often felt proud. |  |
|  | PE5 | During my working, I was often full of inspiration. |  |
| Work alienation  (WA) | WA1 | I do not enjoy my work. | Using with an five-point Likert scale, Variable’s value is calculated on the mean value from these items. |
|  | WA2 | Facing my daily tasks is a painful and boring experience. |  |
|  | WA3 | Work to me is more like a chore or burden. |  |
|  | WA4 | I feel estranged/disconnected from myself. |  |
|  | WA5 | I often wish l were doing something else. |  |
|  | WA6 | Over the years l have become disillusioned about my work. |  |
|  | WA7 | I do not feel like putting in my best effort at work. |  |
|  | WA8 | I do not feel connected to the events in my workplace. |  |
| Perceived algorithmic control  (AC) | AC1 | Algorithms assigns my tasks intelligently. | Using with a five-point Likert scale, Variable’s value is calculated on the mean value from these items. |
|  | AC2 | Algorithms gives specification instructions to my work according to the standards by platform. |  |
|  | AC3 | Algorithms provides me with a lot of information relevant to completing the task. |  |
|  | AC4 | Algorithms gives me real-time feedbacks related to performance. |  |
|  | AC5 | Algorithms tracks and locates my location in real time. |  |
|  | AC6 | Algorithms keeps track of my work. |  |
|  | AC7 | Algorithms monitors my work attitude in real time. |  |
|  | AC8 | Algorithms automatically evaluates the quality of my work. |  |
|  | AC9 | Algorithms classifies my work performance and ranks it within platforms. |  |
|  | AC10 | Algorithms rewards me with cash at certain times to work hard. |  |
|  | AC11 | When I fail to meet a platform's requirements, algorithms fine me. |  |
| Workplace well-being  (WB) | WB1 | I am satisﬁed with my work responsibilities. | Using with a five -point Likert scale, Variable’s value is calculated on the mean value from these items |
|  | WB2 | In general, I feel fairly satisﬁed with my present job. |  |
|  | WB3 | I ﬁnd real enjoyment in my work. |  |
|  | WB4 | I can always ﬁnd ways to enrich my work. |  |
|  | WB5 | Work is a meaningful experience for me. |  |
|  | WB6 | I feel basically satisﬁed with my work achievements in my. |  |
| Gender | Is the respondent male or female? | | “1”=Male,”2”=Female. |
| Age | Age: <=25 years、26 ~35 years、36 ~45years 、>=46 years | | “1”= <25 years,  “2”=26~35years, “3”=36~45years,  “4”= ≥46 years. |
| Education | Education level：High school degree or below、Junior college degree、Bachelor degree、Master degree or above | | “1”=High school degree or below,  “2”=Junior college degree,  “3”=Bachelor degree，  “4”=Master degree or above. |
| Tenure | Tenure: <=1 years、2 ~4 years、5 ~7years、8 ~10years、 >=11 years | | “1”= ≤1 years,  “2”=2~4years,  “3”=5~7years,  “4”=8~10years,  “5”=≥11years. |
| Participate in gig work | How you participate in gig work? | | “1”= Full-time,  ”2”= part-time. |
| Average monthly | Average monthly: <=2000 yuan、2001 ~4000 yuan、4001 ~6000 yuan、6001 ~8000 yuan、>8000 yuan. | | “1”=≤2000 yuan,  “2”=2001 ~4000 yuan,  “3”=4001 ~6000 yuan,  “4”=6001 ~8000 yuan,  “5”=>8000 yuan. |
| Number of children | Number of children: Not raising children, raising a child, raising two child, Raising 3 or more children | | “0”= Not raising children,  “1”= Raising a child,  “2”=Raising two child,  “3”= Raising 3 or more children. |
| Marital status | Your marital status? | | “1”= married,  ”2”= spinsterhood. |
